# Historical and Projected Surface Temperature over India during the 20^th^ and 21^st^ century

**DOI:** 10.1038/s41598-017-02130-3

**Published:** 2017-06-07

**Authors:** Ghouse Basha, P. Kishore, M. Venkat Ratnam, A. Jayaraman, Amir Agha Kouchak, Taha B. M. J. Ouarda, Isabella Velicogna

**Affiliations:** 1 National Atmospheric Research Laboratory, Gadanki, Tirupati India; 2 Department of Earth System Science, University of California, Irvine, California, 92697 USA; 3 Department of Civil and Environmental Engineering, University of California, Irvine, California 92697 USA; 4 Institute Center for Water and Environment (iWATER), Masdar Institute of Science and Technology, P.O. Box 54224 Abu Dhabi, UAE; 5 INRS-ETE, National Institute of Scientific Research, Quebec City (QC), G1K9A9 Canada

## Abstract

Surface Temperature (ST) over India has increased by ~0.055 K/decade during 1860–2005 and follows the global warming trend. Here, the natural and external forcings (e.g., natural and anthropogenic) responsible for ST variability are studied from Coupled Model Inter-comparison phase 5 (CMIP5) models during the 20^th^ century and projections during the 21^st^ century along with seasonal variability. Greenhouse Gases (GHG) and Land Use (LU) are the major factors that gave rise to warming during the 20^th^ century. Anthropogenic Aerosols (AA) have slowed down the warming rate. The CMIP5 projection over India shows a sharp increase in ST under Representative Concentration Pathways (RCP) 8.5 where it reaches a maximum of 5 K by the end of the 21^st^ century. Under RCP2.6 emission scenarios, ST increases up to the year 2050 and decreases afterwards. The seasonal variability of ST during the 21^st^ century shows significant increase during summer. Analysis of rare heat and cold events for 2080–2099 relative to a base period of 1986–2006 under RCP8.5 scenarios reveals that both are likely to increase substantially. However, by controlling the regional AA and LU change in India, a reduction in further warming over India region might be achieved.

## Introduction

Global Surface Temperature (ST) has increased significantly during the last three decades. The recent report of the Intergovernmental Panel on Climate Change (IPCC)^1^ showed the anthropogenic footprint on the global warming of atmosphere and oceans, reduction in snow and ice, and increase in sea level. Climate change and variability comprise complex interactions between natural and external forcings at different temporal and spatial scales. However, quantifying the contribution of each component in the overall observed climate signal is very challenging. Climate models represent important tools to investigate the historical climate change due to anthropogenic forcings. With these models and controlled simulations, it is possible to decompose the contributions from individual factors or assess their combined effects on regional or global climate. This allows exploring the possible causes and understanding the physical mechanisms behind climate change and variability^1^.

The World Climate Research Program (WCRP) has supported the development of the Coupled Model Intercomparison Project (CMIP), which provides simulation from state-of-the-art global climate models. CMIP model simulations have been used in the Assessment Reports of the Intergovernmental Panel on Climate Change (e.g., IPCC AR4 and AR5). The CMIP5 simulations include both historic (20^th^ century) and future projections (21^st^ century) under different Representative Concentration Pathways (RCPs)^2^. The RCPs represent different mitigation scenarios that influence the future emissions of greenhouse gasses, aerosols, ozone and land use change. The CMIP5 experiment simulates historical changes in temperature with forcings driven by Anthropogenic Aerosols (AA), Greenhouse Gases (GHGs), Land Use change (LU), Natural (NAT) forces (Solar Irradiance + Volcanic Activity), and Solar radiation (SL)^3^. Comparing these forcings with each other provides a better understanding of the contributors to climate change over a particular region or location^4–8^.

India is one of the fastest-growing economies in the world. The annual ST has increased since the 1950’s, particularly over India. Although, previous studies^9–11^ have shown a clear increasing trend in mean, minimum, and maximum temperatures over India during the last century, the contributing factors to this have not been explored in detail. The observed climate change in India is due to the complex interactions between natural and anthropogenic forcings at different temporal and spatial scales. However, it is not well understood to what extent climate change in India during the past century, particularly the past several decades, can be attributed to human and natural forcings. In this study, we have utilized the CMIP5 simulation from the years 1860–2100 along with ground-based observational data sets (Climate Research Unit, CRU and India Meteorological Department, IMD gridded temperature) to quantify the historical (Natural + Anthropogenic), NAT, and external forcings (AA, GHGs, LU and SL) in the observed warming over India. We evaluate the 17 CMIP5 historical ST data with observational data sets along with future projections. We investigate the contribution of different natural and anthropogenic forcings in the observed and expected future warming. We also evaluate trends in different simulations during different periods between the years 2006–2100. In addition, we discuss changes in ST extremes over India.

## Results

### Evaluation of CMIP5 ST with CRU and IMD

The ST simulation from the individual CMIP5 model is evaluated against the CRU and IMD data sets as shown in Figures S1 and S2, respectively. These simulations are compared for different periods: 1901–2005 (against CRU data) and 1969–2005 (against IMD data). From both figures, it is clear that the North Eastern part and Himalayan regions show a large bias for all models, possibly due to complexities in higher elevations also mentioned in other studies^12^. Further, the overall bias is even larger when CIMP5 simulations are compared with IMD relative to the CRU data set, mainly on the western coast of India. The models CSIRO-Mk3-6-0, GISS-E2-H, GISS-E2-R, HadGEM2-CC, HadGEM2-ES, MIRCO-ESM, MRI-CGCM3, and inmcm4 show larger biases compared to other models.

**Figure 1:**
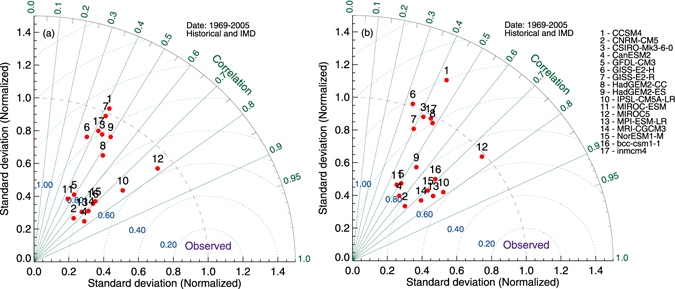
Taylor diagram for mean surface temperature between CRU and CMIP5 individual model during the period 1901–2005 (**a**), IMD and CMIP5 individual model for the years 1969–2005 (**b**) over India. Solid lines correspond to standard deviation; dotted lines for correlations (Figure was created using the Interactive Data Language (IDL) version 8.2 software http://www.harrisgeospatial.com/docs/whats_new_in_82.html).

For further evaluation, we show the Taylor diagrams for representing the simulated mean STs relative to CRU (Fig. 1a) and IMD (Fig. 1b). The former covers 1901–2005, whereas the latter covers 1969–2005 over India. In these figures, the solid lines correspond to standard deviations, while the dotted lines represent the correlations between simulations and observations. As shown, the correlation between 8 CMIP5 models (CCSM4, CSIRO-Mk3-6-0, GISS-E2-H, GISS-E2-R, HadGEM2-CC, HadGEM2-ES, GFDL-CM3, and MICRO-ESM) with CRU and IMD data sets is less than 0.5. To continue the analysis, we have discarded these models, and considered the ones that show high correlations (i.e., CNRM-CM5, CanESM2, GFDL-CM3, IPSL-CM5A-LR, MIRCOC5, MPI-ESM-LR, NorESM1-M, and bcc-csm1-1). The Taylor diagram presents the spread of the models in terms of their spatial correlation. The correlations between models and the two observational data sets are quite similar (Fig. 1a and b). The highest correlation score of about of 0.78 is observed for MIROC5 and IPSL-CM5-AL.

**Figure 2:**
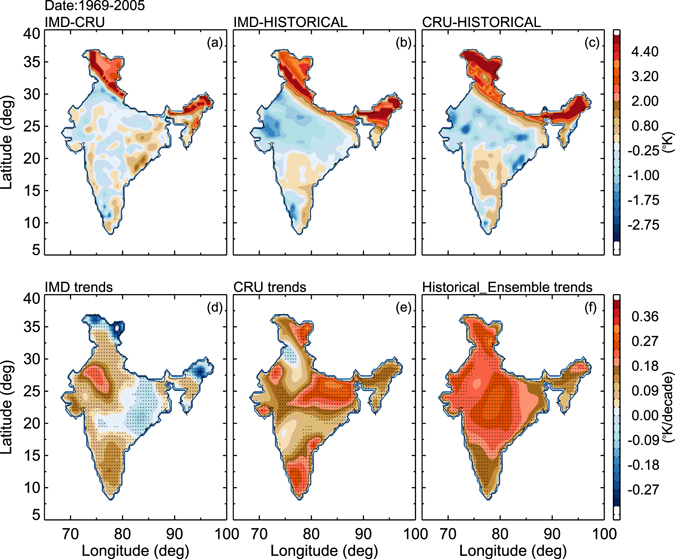
Spatial temperature difference between (**a**) IMD and CRU, (**b**) IMD and ensemble mean of CMIP5 models, and (**c**) CRU and ensemble mean CMIP5 models. Spatial surface temperature trends over India for (**d**) IMD, (**e**) CRU and Ensemble mean of CMIP5 models for the years 1969–2005 with 95% confidence level (hatched regions) (Figure was created using the Interactive Data Language (IDL) version 8.2 software http://www.harrisgeospatial.com/docs/whats_new_in_82.html).

The climatological mean biases between CMIP5 simulations and the two observational data sets (i.e., IMD, CR) are shown in Fig. 2. Note that this bias and trends are estimated for the period of 1969–2005 when the two observational datasets overlap. The mean bias between IMD and CRU is less than ±1 K, as shown in Fig. 2a, except for the Northeastern part and the Himalayan regions. The climatological bias between IMD and the ensemble mean of the selected 8 CMIP5 models is shown in Fig. 2b. The bias over India varies around ±1 K, except over the Northeastern and Himalayan regions where the bias exceeds 4 K. Similar bias is observed between CRU and CMIP5 models. Higher bias in the Northeastern and Himalayan regions may be due to the fact that there are few observations available in these regions. In addition, we computed the spatial trends based on the historical climate model simulations and observations (IMD, CRU) - see Fig. 2(d–f). In Fig. 2(d–f), the solid black mark indicates the 95% confidence level, as determined at each individual grid point and was computed by using student’s t-test. Over India, there is a warming trend at the rate of ~0.2 K/decade from the year 1969–2005. Few parts (Northeastern and Himalayan regions) of India exhibit decreasing trends in IMD data (Fig. 2d). Recent studies also indicate relatively suppressed warming over India, with some cooling over the central regions, during the past century^13^. This feature is not shown in the CRU data and CMIP5 simulations. The central part of India shows the highest warming compared to other regions as shown by both CRU and CMIP5 simulations (~0.36 K/decade). Overall, there are discrepancies in the warming trends. However, CRU observations and CMIP5 simulations seem to be more consistent with one another compared to IMD data. We have also performed the seasonal trend analysis in ST over India as shown in Table 1.

**Table 1:** Historical, CRU and IMD seasonal trends at 95% confidence level over the Indian region.

Name	Seasonal trends (K/decade)				
	Winter	Spring	Summer	Fall	All years
	1860–2005				
Historical	0.056	0.053	0.071	0.040	0.055
Historical_AA	−0.029	−0.025	−0.019	−0.033	−0.027
Historical_GHG	0.121	0.092	0.115	0.099	0.106
Historical_LU	0.086	0.054	0.079	0.074	0.073
Historical_NAT	0.019	0.025	0.012	0.004	0.006
Historical_SL	−0.014	−0.004	−0.015	0.005	−0.007
	**1901–2005**				
Historical	0.077	0.069	0.100	0.489	0.074
CRU	0.084	0.022	0.064	0.072	0.060
	**1969–2005**				
Historical	0.225	0.183	0.217	0.199	0.207
IMD	0.137	0.112	0.068	0.089	0.081
CRU	0.252	0.116	0.099	0.202	0.168

### Impact of different forcings on ST

**Figure 3:**
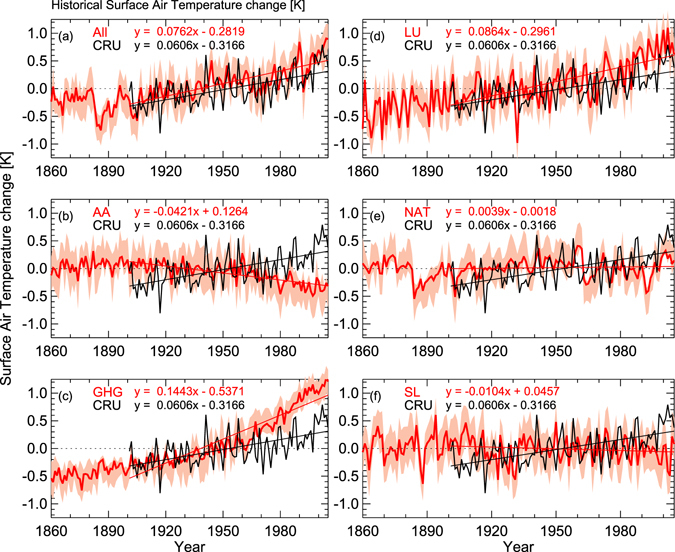
Temporal variation of annual surface temperature anomalies during the years 1860–2005. ‘All’ indicates the historical forcing, which contains all types of forcings. Individual forcings include Anthropogenic Aerosol (AA), Greenhouse Gases (GHG), Land Use change (LU), Natural forcings (solar radiation + volcanic eruptions), and Solar radiation (SL). In each forcing the observed CRU temperature data is also plotted for the years 1901–2005. Robust regression analysis was performed at the 95% confidence level for trend analysis which is over plotted in each panel (Figure was created using the Interactive Data Language (IDL) version 8.2 software http://www.harrisgeospatial.com/docs/whats_new_in_82.html).

The ensemble means of different historical forcings for the 19^th^ and 20^th^ centuries (shown in Table S1) are plotted in Fig. 3. The forcing responses are computed and compared with the observed ST based on ground-based data. Figure 3 show temporal variation of mean ST over India from CRU, and historical simulation (GHG, NAT, AA, LU, and SL forcings) data. These temporal variations are calculated as the mean of all years over India (using data from Jan. 1860 to Dec. 2005) subtracted from the yearly ST values of each simulation. In each panel, the ensemble bounds (maximum and minimum values) are shown in shaded color. The results of the robust regression fitting, along with CRU ST are shown in Fig. 3 for each forcing. The increase in temperature due to GHGs forcing from 1900 onwards is clearly observed in Fig. 3b. The turning point (changing from negative to positive temperature anomaly) in the yearly time series of ST variation matches very well with the historical ensemble mean of the different forcings. The observed CRU ST is superimposed on the ensemble of historical model simulations (Fig. 3a). Both historical simulations and CRU ST show similar variability throughout the 20^th^ century (Fig. 3a). Except for NAT, SL and AA, all other forcings show a remarkable increase in ST, some even higher than the observations (e.g., 3c and 3d). This shows that considering LU and GHG alone leads to an overestimation of the surface temperature warming in the historical period. However, increase in ST from the combination of all forcings closely follows the observed historical temperatures. The warming due to GHG shows a sharp increase after1960 over India, which is very much consistent with LU forcings. The combination of both direct and indirect AA forcings depicts the significant decreasing from 1960 onwards. The impacts of NAT and SL forcings on warming in ST are comparatively much smaller than those from GHG, and LU. These simulations with individual forcings are useful to compare the role of different forcings. Individually, they are not expected to reproduce historical observations. However, we have included the historical observations as a common reference for evaluation. Overall, the results show that AA, NAT and SL provided cooling effect and slow the warming rate, while the other forcings (GHG and LU) contributed significantly to the observed warming. The results show that AA has negative contribution to the warming when we considered both the direct and indirect forcings. When direct forcings alone is consider, there is a weak cooling trend. However, with both direct and indirect AA the cooling trend becomes highly significant. Under GHG forcings, only the warming trend of 0.16 K/decade is observed which is very strong compared to all other forcings. The seasonal variations of different forcings are depicted in Table 1. The largest seasonal variations in GHG and LU are observed during winter followed by summer, fall and spring. GHG variability is larger during winter and summer compared to the other seasons. The observed increasing trend is larger for GHG compared to LU. In case of AA forcings, the seasonal variation of decreasing trend is large during fall and minimum during summer. A small positive trend is noticed from NAT forcing in all the seasons. The SL radiation shows a significant negative trend, except during the fall season.

**Figure 4:**
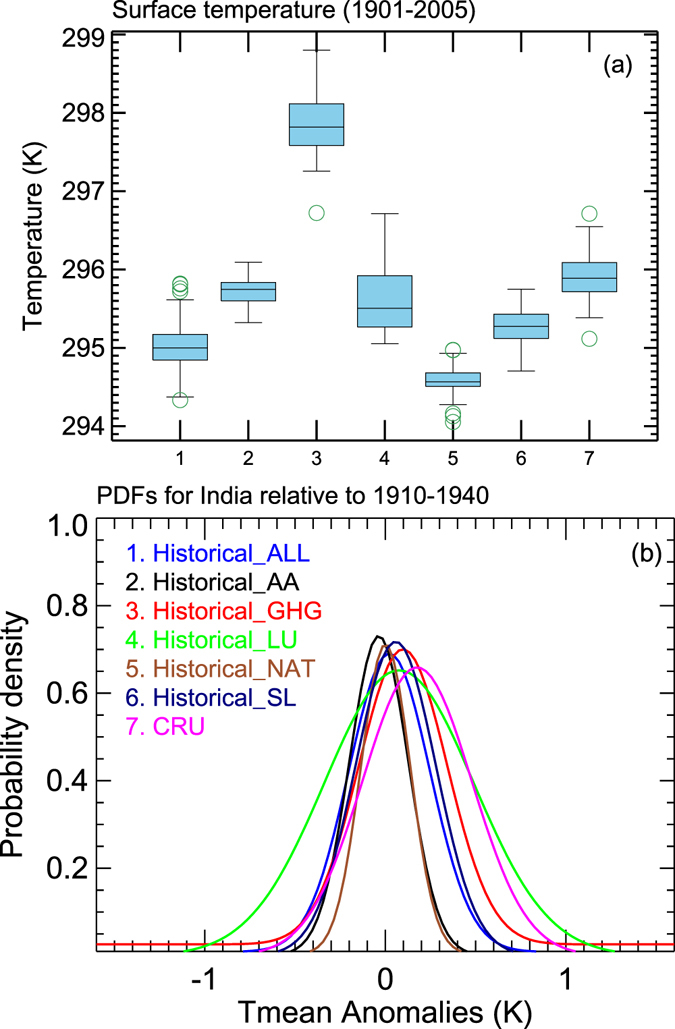
(**a**) Box-and-whisker plots for temperature of different forcings along with observed CRU data sets from 1901–2005. The boxes indicate the interquartile model spread (range between the 25th and 75th quantiles), the black/grey solid marks within the boxes show the multimodel median and the whiskers indicate the total intermodel range. (**b**) Probability density distribution of surface temperature over the Indian region relative to the years 1910–1940 for different forcings (Figure was created using the Interactive Data Language (IDL) version 8.2 software http://www.harrisgeospatial.com/docs/whats_new_in_82.html).

Figure 4 shows the box and whisker plot along with probability density functions of ST from different forcings relative to the base period of 1910–1940. The boxes indicate the interquartile model spread (i.e., 25^th^ and 75^th^ quartiles). The 75^th^ quartile of the GHG forcing is highest among others and reaches 298.2 K. Figure 4 shows that the difference between GHG and NAT forcings is around ~3 K, which suggests the role of GHG alone relative to the natural variability. The highest ST values are found in GHGs followed by LU, AA, SL and NAT, respectively. The probability density functions of the historical temperature anomalies (relative to the period 1910–1940) show clear warming shifts of about ~0.15 K in GHGs, followed by LU. The AA forcings shows the negative shift in the probability distribution. The distributions of the remaining forcings fall between 0–0.2 K temperature changes in the historical period^14^. Showed the competing roles of GHG and AA with regard to Indian Ocean warming.

**Figure 5:**
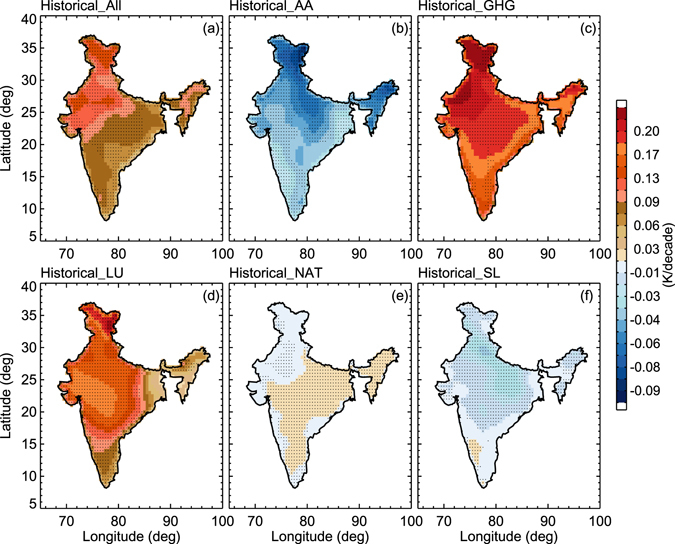
Geographical distribution of surface temperature trends (K/decade) for historical data along with different forcings. (**a**) Historical surface temperature, (**b**) AA, (**c**) GHG, (**d**) LU, (**e**) NAT, (**f**) SL. Trends were estimated at the 95% confidence level (hatched regions) for each grid (Figure was created using the Interactive Data Language (IDL) version 8.2 software http://www.harrisgeospatial.com/docs/whats_new_in_82.html).

The spatial distribution of ST trends over India is estimated using 146 years of simulations (1860–2005) with different forcings (Fig. 5). Here, the ST trends are computed at each grid point using the robust regression technique^12^. In this process, we have tested the statistical significance at each grid cell and only considered trends that are significant at the 0.05 significance level (95% confidence) marked with ‘x’ symbol. Figure 5a shows the warming response over India at a 0.18 K/decade rate based on all forcings. The NAT forcings show a slight warming trend over central India and cooling trends in other parts, which, as expected, is not consistent with the observations. The response of GHGs and LU forcing is very large and a warming trend is observed throughout the region. The warming (cooling) rate response to GHG (AA) forcing in North India is large compared to the South India (Fig. 5b and c).

Sensitivity experiments, such as correlation analysis with all forcings, are also performed in the present work. The correlation values are presented in Table S2. The observed ST from CRU shows good correlation with GHG forcings while historic data shows a very high correlation with GHG and LU. A negative correlation is detected between AA and SL with observed data. GHG emissions contributed to a warming of about 0.106 K/decade over the period 1860 to 2005 whereas the contributions LU to the warming trend are around 0.073 K/decade. The contribution from natural forcings is about 0.006 K/decade, very much less than external forcings. Solar radiation shows a negative trend, which means a cooling effect on ST. The contribution of natural and external forcings contributed to a mean surface warming of about 0.055 K/decade. It is extremely likely that more than half of the observed increase in average ST from 1950 onwards was caused by external forcings.

### Future projections

**Figure 6:**
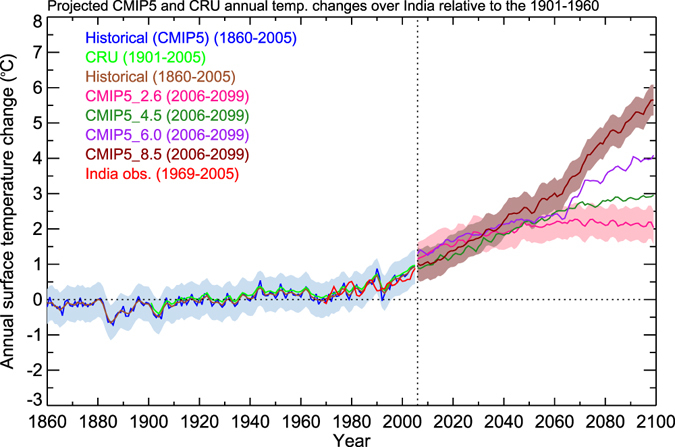
Projected annual mean surface temperature from multimodel average CMIP5 data for the years 1860–2100 relative to the base period 1901–1960. The period 2006–2099 represents the future projection scenarios from different RCPs. The shaded region represents the standard deviation of the ensemble mean of CMIP5 models. The observed data sets CRU (1901–2005) and IMD (1969–2005) are also plotted (Figure was created using the Interactive Data Language (IDL) version 8.2 software http://www.harrisgeospatial.com/docs/whats_new_in_82.html).

For projecting climate change impacts on ST during the 21^st^ century, we considered low and high RCPs (i.e., RCP2.6 and RCP8.5) emission scenarios. The evolution of ST changes relative to 1901–1960 over India from IMD, CRU, historic simulations, and ensemble mean of 8 CMIP5 simulations are shown in Fig. 6. RCP2.6 and RCP8.5 show around 3.2 degrees temperature difference by the end of the century. During the last century, the change in surface temperature is well captured by all data sets. Temperature projections show consistent increasing trends in RCP8.5 whereas the projected mean temperature from RCP2.6 increases up to 2050, and then slightly decreases, consistent with the radiative forcing under RCP2.6. The mean temperature over India is expected to increase over the 21^st^ century under all the RCPs. Around the middle of 21^st^ century, the rate of warming increases sharply depending on the scenario (e.g., RCP 8.5). On average, the projected change in temperature by the end of the 21^st^ century relative to 1901–1960 is expected to be 1.8 K, 2 K, 3.5 K, and 5 K for RCP2.6, RCP4.5, RCP6.0 and RCP8.5 emission scenarios, respectively.

**Figure 7:**
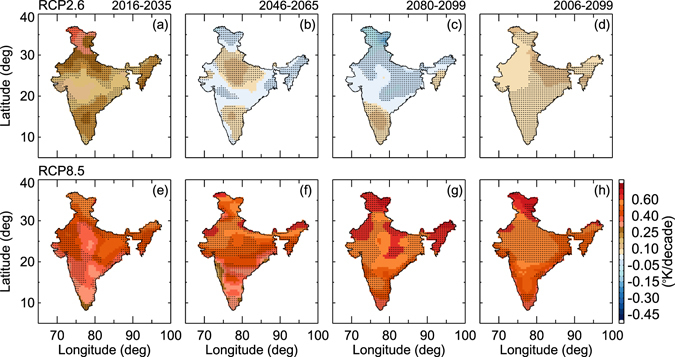
Geographical distribution of surface temperature trends (K/decade) at the 95% confidence level (hatched regions) from RCP2.6 and RCP 8.5 for different periods over India for the 21^st^ century. Different periods correspond to near (2016–2035), mid (2046–2065) and far (2080–2099) and for the whole period (2006–2099) (Figure was created using the Interactive Data Language (IDL) version 8.2 software http://www.harrisgeospatial.com/docs/whats_new_in_82.html).

Figure 7 illustrates the spatial pattern of ST trends over India during the 21^st^ century under RCP2.6 and RCP8.5 emission scenarios. The ST trends were estimated for different periods such as near future (2006–2035), mid-future (2046–2065), and far future (2080–2099) as well as for the entire time series of model simulations (2006–2099). Under RCP2.6, the temperature increases in near future and decreases in both mid- and far-future, but the entire time series shows an overall increase in ST of about ~0.15 K/decade. The far-future projections show cooling all over India except for southern India (Fig. 7c). Continuous increases in ST are expected in near, mid-, and far future under the RCP8.5 scenario. Significantly, higher temperatures are expected in the late 21^st^ century compared to near and middle future temperatures. The long-term (2006–2099) trends show warming of about 0.2 K/decade over India. In addition, the warming increases rapidly toward the end of the 21^st^ century, reaching a peak value of about 0.4 K/decade.

We have also analyzed the seasonal variations of ST trends over India from RCP2.6 and RCP8.5 as shown in Table S3 for different time periods. In the near future, under RCP2.6 the temperature increases sharply and is highest during the summer season, while ST decreases in mid- and far-future. Under RCP8.5, the ST increases during all periods. The far-future ST trend increases drastically (nearly 3 times the near and mid-future trends). The winter ST shows significant increase followed by spring over India. The entire time series indicates that ST can increase by ~4.5 K by the end of the 21^st^ century.

### Extreme Temperatures

Recent studies have shown the variability and mechanisms for occurrence of heat waves over India^15, 16^. Increasing trends in frequency, total duration and maximum duration of heat waves are observed over the central and northwestern parts of India^13^. showed that the increase in heat wave activity is due to global warming over India. Here, the extremely high and low monthly temperatures (i.e. rare heat and rare cold events) from CMIP5 simulations are analyzed. The 20-year difference value of the annual maximum or minimum temperature is a measure of changes in rare temperature extremes^17, 18^. Figure S3 indicates the projected change in the 20-yr return value of the annual maximum and minimum daily ST over India by the end of the 21^st^ century (2081–2100), relative to the recent past (1986–2005) from RCP2.6 and RCP8.5 emission scenarios. The annual maximum temperature extremes under RCP2.6 are projected to decrease by the end of the 21^st^ century, except for the Eastern part of central India and the Himalayan region. Under RCP8.5, the Himalayan region is projected to face higher extreme temperatures by the end of the 21^st^ century. Moderate increases are expected in the Northeastern, Central and Northwestern parts of India (Figure S3 top right panel). Figure S3 (bottom) shows the minimum temperature extremes (rare cold events) under RCP2.6 and RCP8.6 emission scenarios. The projected changes in rare cold events are higher under RCP8.5 than RCP 2.6. The northern part of India can expect larger changes than the other parts of the country.

## Summary and Conclusions

The surface temperature (ST) changes over India are studied by using 109 simulations from global coupled climate models with different external forcings during the 20^th^ and future projections under different RCP emission scenarios in the 21^st^ century. Based on the CMIP5 multi-model simulations, the relative contribution of external forcings such as natural (solar radiation, volcanic emissions) and human activities (GHGs, AA, and LU) to the observed ST during the last 100 years were computed. In addition, we have also considered the individual and combined effects of direct and indirect AA forcings. The evaluation of CMIP5 historic data with observational data sets and future projections during the 21^st^ century under different RCP scenarios is also presented. The long-term trends were estimated by using robust regression analysis. The main findings of the present study are summarized below:

- During the 20^th^ century, the major contributors for the increase in ST are GHGs and LU over India whereas the AA has slowed the warming rate over India. The NAT and SL forcings shows slight negative warming trend. The warming rate from GHGs and Lu are estimated to be 0.14 ± 0.53 and 0.06 ± 0.2 K/century, respectively.
- The AA forcings show cooling effect when we considered both the direct and indirect forcings. The cooling effect is weekend when only direct effect of AA is considered.
- Human activities are highly responsible for the increase in warming over India. The warming is predominantly attributed to GHGs followed by LU. A sharp increase in warming is noticed from the 1960s onward from GHGs, whereas for LU the increase is observed from 1980^19, 20^.
- The spatial variation of trends from different forcings over India indicates that the impact of GHGs is more severe over the Northern and Western parts of India.
- The trends estimated during the 20^th^ century show highest warming in historic data followed by CRU and IMD data. The central part of India warmed severely compared to other regions. The observed warming is more pronounced during the summer followed by winter, monsoon season, and post-monsoon.
- The multi-model mean of CMIP5 captures the basic features of observed ST. The future protections under different RCP emission scenarios are very distinct. Rapid increase in warming is noticed under RCP8.5 where it reaches a maximum temperature of ~5 K by the end of the 21^st^ century. In RCP2.6 emission scenarios the warming increases until 2050 and decreases afterwards.
- Future warming is larger during the winter season followed by summer, monsoon and post-monsoon seasons over India. By the end of the 21^st^ century, both rare cold and heat events are expected to increase over India under RCP 8.5 emission scenario.

GHGs (water vapor, CO_2_, CH_4_, N_2_O) act to make the surface warmer by absorbing and emitting heat energy in all the directions. Addition of more GHGs to the atmosphere make more effective in preventing the heat from escaping into space. The GHG’s concentration increased significantly since the industrial revolution began. The atmospheric concentrations of CO_2_, CH_4_, N_2_O has increased by 40%, 150% and 20%, respectively. This increase in GHGs is due to emissions by human activities, which alter the Earth’s energy balance resulting in significant increase in ST thus global warming. The anthropogenic forcings plays a significant role in climate change hence global warming. Humans alter aerosols not only at the surface (e.g. industrial emissions) but also at the higher altitudes. Due to increase in populations, the natural vegetation decreases for thousands of years by deforestation. The largest changes occurred in 20^th^ century. Hence changing land cover affects the climate by modifying the surface reflectivity and the hydrological cycle. In addition to the direct radiative effects, LU can affect the sources and sinks of GHG’s and the amount of dust lifted into the atmosphere by the wind. For example, atmospheric CO_2_, CH_4_, and N_2_O concentrations are increased by deforestation, cultivation of rice and by use of nitrogen fertilizers in agriculture activity. Natural variability of aerosols, particularly due to volcanoes eruptions (e.g. Mt. Pinatubo in 1991) are recognized as a significant climate change indication, which alters the Earth’s radiation balance and thus tends to cause the increase in temperature. These and other lines of evidence point conclusively to the fact that the elevated CO_2_ concentration in our atmosphere is the result of human activities. It is well known that net global warming is very sensitive to the region of focus. Different local and regional topographical and meteorological factors play an important role in determining global warming and climate change. India will observe changes in precipitation, cyclone occurrence, human health, agriculture, extreme events^21^. Although a sudden increase in ST was observed during the last century, the annual precipitation over India showed only moderate increase. However, the extreme precipitation frequency and magnitude increased rapidly^22, 23^. Nevertheless, newly introduced forcing agents such as historical emissions of carbonaceous aerosols should be included in future studies for a better understanding of global warming impacts. Uncertainties related to future warming in model analysis also need further investigation. CMIP5 coordinated climate models experiments had leads to better understanding of past, present, and future climate change and variability. However, the science gaps and outstanding questions have promoted to design new experiments known as CMIP6, which address the science aspects such as Earth system respond to forcings, sources and consequences of systematic biases and assessment of future climate changes given climate variability, predictability and uncertainties in scenarios. In recent years, the asymmetry of the forcing has been noticed and attracted more and more attention of scientists. For example, the asymmetric volcanic eruptions at different latitudes can affect the global climate differently^24^. In CMIP6, the latitude-dependent GHG concentration will also considered, that should have different effect on the Indian surface temperature compared to CMIP5.

## Data and Methodology

### Historical and Future projections from CMIP5 models

In this article, we have analyzed 109 simulations from 17 CMIP5 models, which include different emission scenarios, and historical simulations as shown in Table S1. Note that not all CMIP5 models simulate all the forcings. 17 CMIP5 models under different RCP 2.6, 4.5, 6.5, 8.5 emission scenarios are used along with NAT forcings from 12 simulations, GHGs forcings from 15 simulations, AA forcings from 5 simulations, LU forcings from 5 simulations and SL forcings from 4 simulations. The period 1860–2005, which is designated as the historic period, has been chosen for coordinated climate model experiments under CMIP5^2^. In this simulation, varying atmospheric composition (e.g., CO_2_) resulting from solar forcing, anthropogenic, volcanic, emissions of short-lived species and natural and anthropogenic aerosols are forced to reproduce the historical climate. These experiments are part of the CMIP5 coordinated experiments, used in the fifth Assessment Report by IPCC^1^. In this study, we used multiple ensembles from 17 CMIP5 models with monthly mean ST outputs available for all four scenarios (RCP2.6, RCP4.5, RCP6.0, and RCP8.5)^25^, and historical simulations (NAT, AA, GHGs, LU, and SL) with different forcing (see Table S1). The RCP4.5 corresponds to radiative forcing of 4.5 Wm^−2^ after the year 2100, which means that the CO_2_ emissions may exceed 650 ppm. Similarly, the RCP8.5 corresponds to 8.5 Wm^−2^ after the year 2100, which means that the equivalent CO_2_ exceeds 1370 ppm.

### Observational data sets

#### CRU data

The monthly gridded ST data from Climate Research Unit (CRU) version 3.22 is used in the present study for the years 1901–2005. This data is available with 0.5° × 0.5° latitude/longitude grid over land regions only over the whole globe. This data is produced by CRU at the University of East Anglia making use of more than 4000 weather station data globally^26^.

#### IMD gridded data

The India Meteorological Department (IMD) developed gridded ST data at 1° × 1° resolution^27^. The observed data from 395 stations were selected for the development of gridded data. The observed data was subjected to quality checks such as rejecting values, which exceed known extreme levels, maximum temperature less than minimum temperature, same temperature values for many consecutive days etc. The 395 station data were interpolated into grids with the modified version of Shepard angular distance weighting algorithm^28^. The data period spans between the years 1969–2005 and it is cross validated by estimating the root mean square error. The errors associated with the interpolation scheme were used in preparing gridded data over the plains, which was found to be 0.5 °C at the maximum.

#### Methodology

The ST data from different forcings and all the CMIP5 model data sets under various RCPs are brought to a common grid of 1° × 1° longitude and latitude by using cubic spline interpolation. For trend estimation, the robust regression technique is used. This method is an important tool for analyzing the data affected by outliers and it is based on Iteratively Reweighted Least Squares Regression (IRLS)^29^. Robust regression is used to detect and provide results that are resistant to outliers^30, 31^. The t-test analysis is employed to calculate the statistical significance of the temperature trends. In this process, we have estimated the confidence level at each trend grid point and only those spectral peaks with greater than 95% confidence level are considered.

## Electronic supplementary material

Supplementary information
