# Supplementary material for: Historical and Projected Surface Temperature over India during the 20th and 21st century
Source: Sci Rep. 2017 Jun 7;7:2987. doi: 10.1038/s41598-017-02130-3 (PMC5462738; doi:10.1038/s41598-017-02130-3)
Supplement: Supplementary file 1 — Supplementary information [file 41598_2017_2130_MOESM1_ESM.doc]

**Historical and Projected Surface Temperature over India during the 20^th^ and 21^st^ century**

**Ghouse Basha^1*^, P. Kishore^2^, M. Venkat Ratnam^1^, A. Jayaraman^1^, Amir AghaKouchak^3^, Taha B.M.J. Ouarda^4, 5^, and Isabella Velicogna^2^**

^1^National Atmospheric Research Laboratory, Gadanki, Tirupati, India.

^2^Department of Earth System Science, University of California, Irvine, California, 92697, USA.

^3^Department of Civil and Environmental Engineering, University of California, Irvine, California, 92697, USA.

^4^Institute Center for Water and Environment (iWATER), Masdar Institute of Science and Technology, P.O. Box 54224, Abu Dhabi, UAE.

^5^INRS-ETE, National Institute of Scientific Research, Quebec City (QC), G1K9A9, Canada.

Correspondence to Ghouse Basha (mdbasha@narl.gov.in)


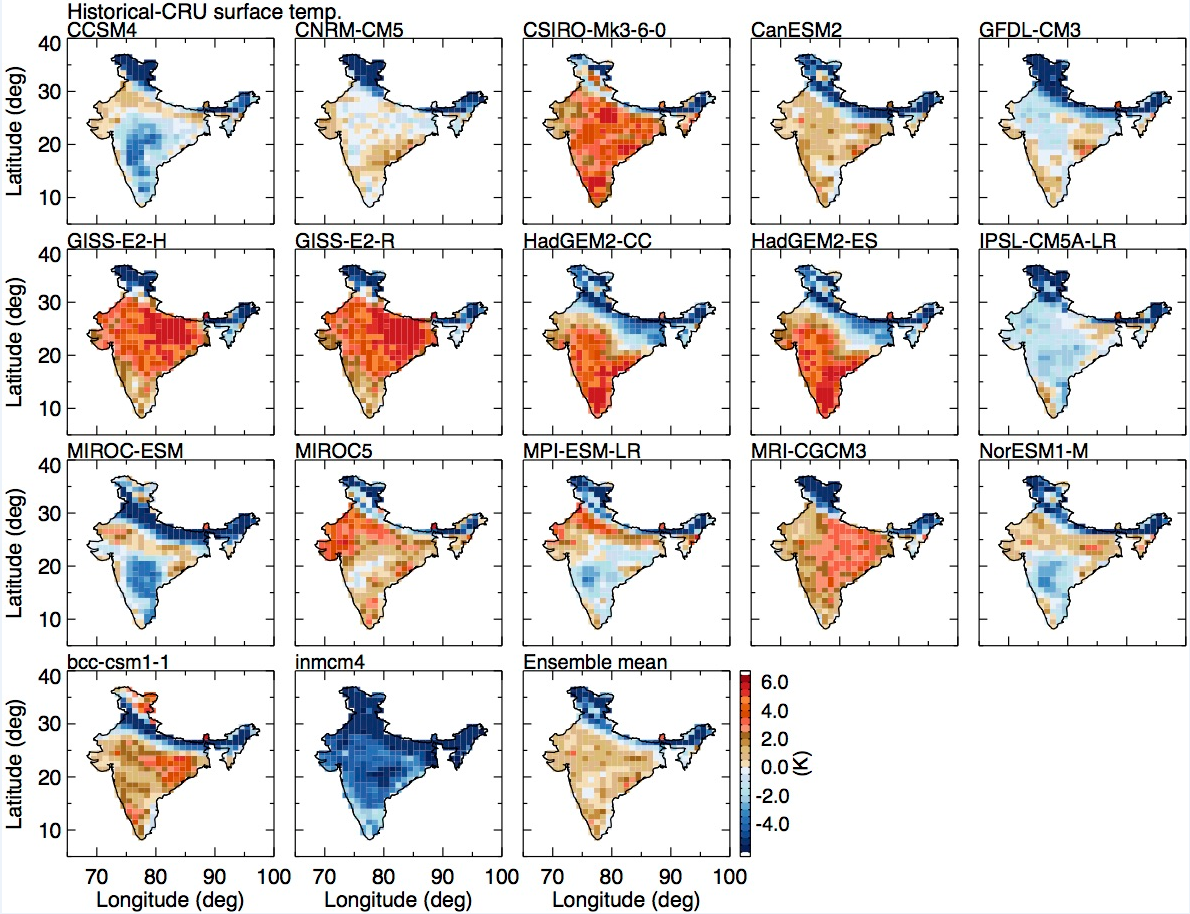


Figure S1. Climatological mean difference between CRU and individual surface temperature data for 1901-2005 along with ensemble mean difference (Figure was created using the Interactive Data Language (IDL) version 8.2 software, http://www.harrisgeospatial.com/docs/whats_new_in_82.html).


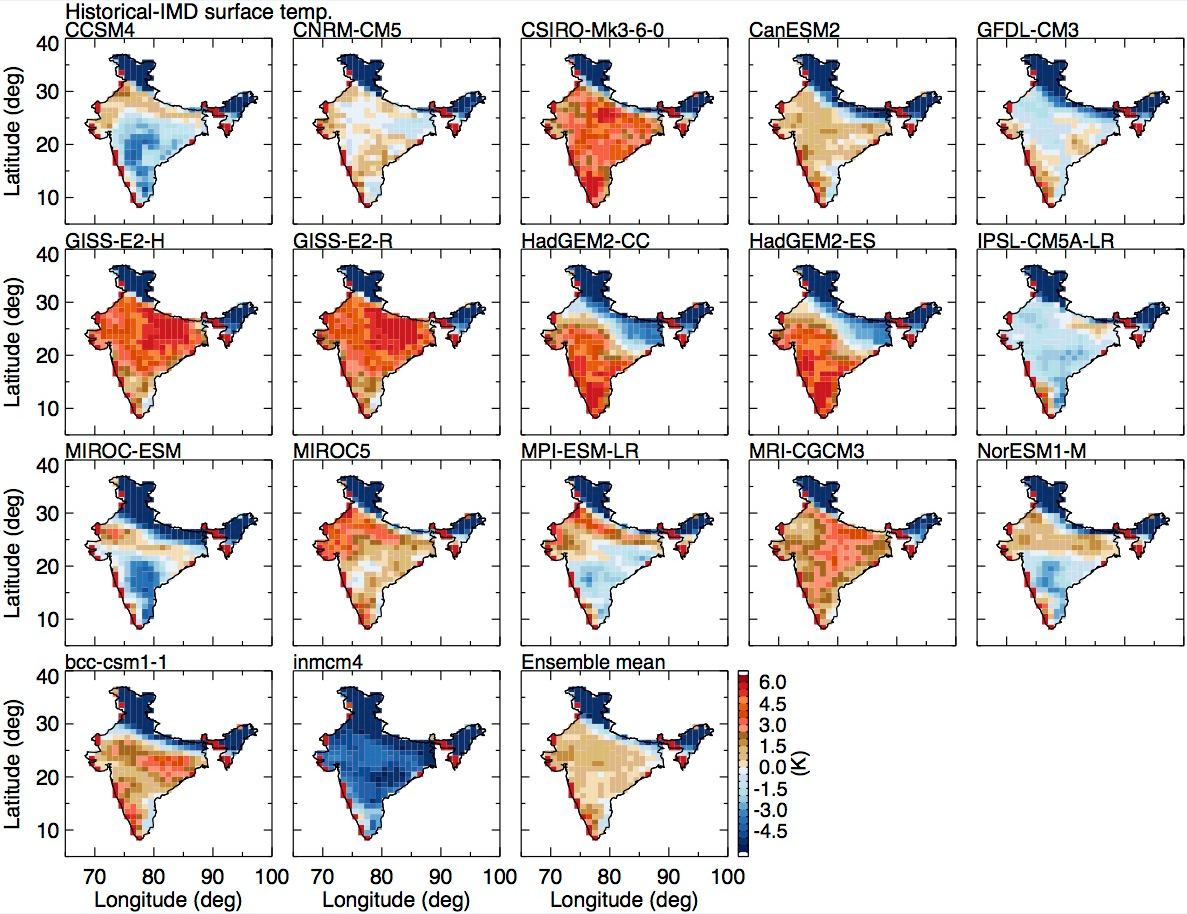
Figure S2. Climatological mean differences between IMD and individual simulation of surface temperature data from 1969-2005 along with ensemble mean difference (Figure was created using the Interactive Data Language (IDL) version 8.2 software, http://www.harrisgeospatial.com/docs/whats_new_in_82.html).


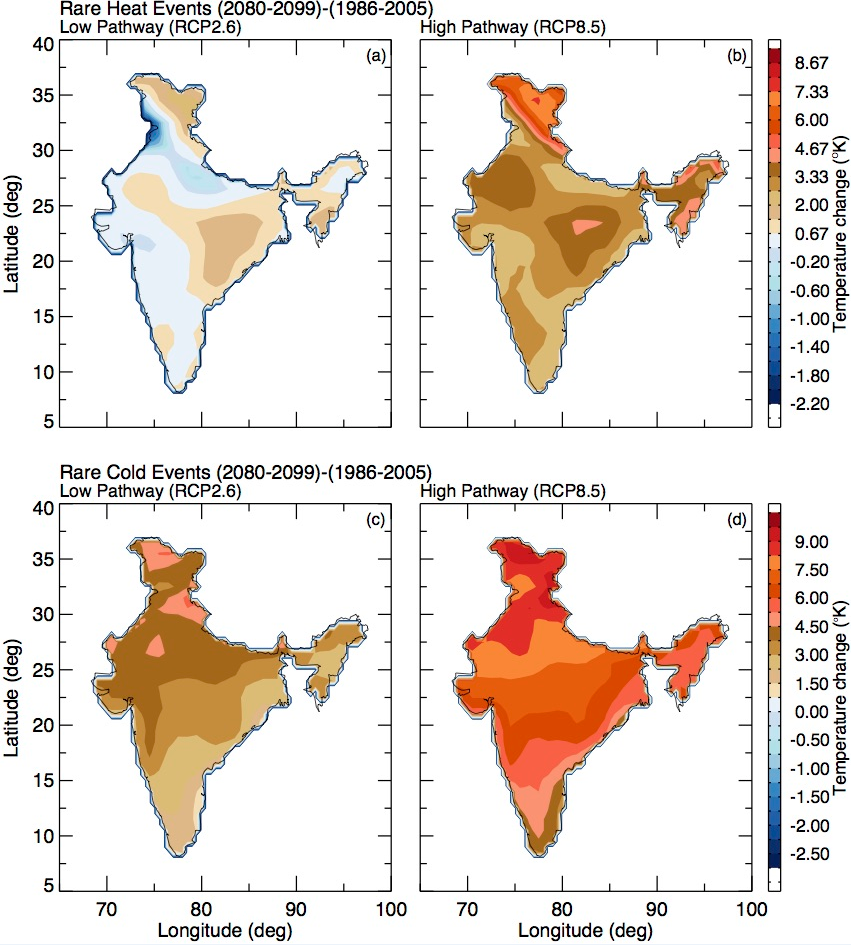


Figure S3. Projected changes in annual maximum and minimum surface temperature from RCP2.6 and RCP 8.5 relative to recent past (1986-2005) over India (Figure was created using the Interactive Data Language (IDL) version 8.2 software, http://www.harrisgeospatial.com/docs/whats_new_in_82.html).

Table S1. Sixty-eight of CMIP5 simulations of RCP26, RCP45, RCP60 and RCP85 scenarios are used in our study. The forcing used for the historical simulations (Y indicates the forcing was used in this simulation and N indicates the simulation is not available). AA: anthropogenic aerosols, GHG: well-mixed greenhouse gases, LU: land-use change, NAT: natural forcing, SL: solar irradiance. (All forcing information was gathered from the CMIP5 website: http://cmip-pcmdi.llnl.gov/index.html.)

| **Model Name** | **CMIP5 2006-2099** | | | | **Historical 1860-2005** | | | | |
| --- | --- | --- | --- | --- | --- | --- | --- | --- | --- |
|  | **RCP2.6** | **RCP4.5** | **RCP6.0** | **RCP8.5** | **AA** | **GHG** | **LU** | **NAT** | **SL** |
| CCSM4 | Y | Y | Y | Y | Y | Y | Y | Y | Y |
| CNRM-CM5 | Y | Y | Y | Y | N | Y | N | Y | N |
| CSIRO-MK3 | Y | Y | Y | Y | N | Y | N | N | N |
| CanESM2 | Y | Y | Y | Y | N | Y | Y | Y | Y |
| GFDL-CM3 | Y | Y | Y | Y | Y | Y | N | Y | Y |
| GISS-E2-H | Y | Y | Y | Y | Y | Y | Y | Y | Y |
| GISS-E2-R | Y | Y | Y | Y | Y | Y | Y | Y | N |
| HadGEM2-CC | Y | Y | Y | Y | N | N | N | N | N |
| HadGEM2-ES | Y | Y | Y | Y | N | Y | N | Y | N |
| IPSL-CM5A-LR | Y | Y | Y | Y | Y | Y | N | Y | N |
| MIROC-ESM | Y | Y | Y | Y | N | Y | N | Y | N |
| MIROC5 | Y | Y | Y | Y | N | N | N | N | N |
| MPI-ESM-LR | Y | Y | Y | Y | N | N | N | N | N |
| MRI-CGCM3 | Y | Y | Y | Y | N | Y | N | N | N |
| NorESM1-M | Y | Y | Y | Y | N | N | N | Y | N |
| bcc-csm1-1 | Y | Y | Y | Y | N | Y | N | Y | N |
| inmcm4 | Y | Y | Y | Y | N | N | N | N | N |
| GFDL-ESM2M | N | N | N | N | N | Y | Y | N | N |
| BNU-ESM | N | N | N | N | N | Y | N | Y | N |
| IPSL-CM5A-MR | N | N | N | N | N | Y | N | Y | N |
| FGOALS_g2 | N | N | N | N | Y | N | N | N | N |
| Total | 17 | 17 | 17 | 17 | 4 | 15 | 5 | 12 | 4 |

Table S2. Correlation between observed data and different forcings of CMIP Historical models

|  | CRU | ALL | AA | GHG | LU | NAT | SL |
| --- | --- | --- | --- | --- | --- | --- | --- |
| CRU | 1.0 | 0.63 | 0.58 | 0.72 | 0.48 | 0.35 | -0.20 |
| ALL | 0.63 | 1.0 | -0.62 | 0.88 | 0.84 | -0.06 | -0.18 |
| AA | -0.48 | -0.62 | 1.0 | -0.78 | -0.67 | 0.06 | 0.23 |
| GHG | 0.72 | 0.88 | -0.78 | 1.0 | 0.75 | 0.19 | -0.12 |
| LU | 0.48 | 0.84 | -0.67 | 0.75 | 1.0 | 0.28 | -0.10 |
| NAT | 0.35 | -0.06 | 0.06 | -0.19 | 0.28 | 1.0 | -0.13 |
| SL | -0.20 | -0.18 | 0.23 | -0.12 | -0.10 | -0.13 | 1.0 |

Table S3. Future projections of seasonal trends during different periods and for the whole period over Indian region using different Representative Concentration Pathways (RCPs).

| Years (K/decade) | 2006-2030  RCP 2.6 (8.5) | 2046-2065  RCP 2.6 (8.5) | 2066-2099  RCP 2.6 (8.5) | 2006-2099  RCP 2.6 (8.5) |
| --- | --- | --- | --- | --- |
| Winter | 0.28 (0.45) | 0.02 (0.48) | -0.03 (0.74) | 0.10 (0.58) |
| Summer | 0.35 (0.35) | 0.04 (0.50) | -0.03 (0.69) | 0.09 (0.56) |
| Monsoon | 0.28 (0.27) | -0.03 (0.21) | -0.03 (0.60) | 0.09 (0.43) |
| Post monsoon | 0.25 (0.37) | 0.14 (0.32) | -0.04 (0.61) | 0.09 (0.50) |
| Annual | 0.29 (0.36) | 0.05 (0.38) | -0.03 (0.66) | 0.09 (0.52) |
